# Diet, physical activity, and UV protection comprehensively influenced vitamin D status in college students: a cross-section study from China

**DOI:** 10.1186/s41043-023-00421-2

**Published:** 2023-07-26

**Authors:** Yingyi Luo, Chunbo Qu, Rui Zhang, Jingyi Zhang, Dan Han, Qingwen Zhang, Jiaxing Li, Lixin Na

**Affiliations:** 1 Medical Technology College, Shanghai University of Medicine and Health Sciences, 279 Zhouzhu Road, Pudong New Area, Shanghai, 201318 China; 2 Public Health College, Shanghai University of Medicine and Health Sciences, 279 Zhouzhu Road, Pudong New Area, Shanghai, 201318 China

**Keywords:** Serum 25(OH)D_3_, Vitamin D deficiency, Dietary intake, Physical activity, UV protection

## Abstract

**Background:**

Vitamin D deficiency is one of the most prevalent health problems worldwide in all age groups, whereas vitamin D status of Chinese college students was seldom studied in China. The purpose of this study was to explore the vitamin D status in Chinese college freshmen and its influencing factors, providing evidence for nutrition strategy application.

**Methods:**

Information including demographic status, diet habit, physical activity, and ultraviolet ray (UV) protection was collected by online questionnaire. Serum 25(OH)D_3_ concentrations were measured using a liquid chromatograph mass spectrometer. Multivariate linear regression analyses were used to explore the comprehensive influence of diet, physical activity and UV protection on serum 25(OH)D_3_ levels.

**Results:**

Totally 1667 freshmen from 26 provinces, autonomous districts or municipalities, were recruited, with a mean age of 18.6 ± 0.9 years. The mean serum 25(OH)D_3_ levels were 18.1 ± 6.3 ng/mL and the proportion of vitamin D deficiency and insufficiency was 67.5% and 27.8%, respectively. Multivariate linear regression indicated that higher intake of milk and yogurt, calcium or vitamin D supplementation, and longer time of outdoor activity were positively linked to higher serum 25(OH)D_3_, while higher intake of candy and higher UV protection index were negatively associated with serum 25(OH)D_3_, after adjusted for age, gender, region of original residence, latitudes, longitude and BMI.

**Conclusions:**

Vitamin D deficiency is very common in Chinese college students. Milk and yogurt intake and outdoor activity should be encouraged while candy intake should be limited for preventing vitamin D deficiency. Public health policies should focus on these changeable lifestyles and consider well-balanced guidelines on UV protection and vitamin D supplementation.

## Background

Vitamin D has been found not only plays an important role in skeletal health, but also protect against cancer, autoimmune diseases, cardiovascular disease, metabolic syndrome (MetS), infections and etc. [1–5]. The multiple health benefits of vitamin D have drawn more and more attention in nutritional strategies for disease prevention.

However, vitamin D deficiency is still a major public health problem worldwide, especially in children, pregnant women and old people [6–8]. A study from Japan indicated that vitamin D deficiency was 73.2% in Japanese pregnant women and the study from China indicated that the prevalence of Vitamin D deficiency and insufficiency among elderly Chinese individuals were 41.3% and 35.7%, respectively [3, 9].The results from the Chinese National Nutrition and Health Survey (CNNHS) 2010–2012 indicated the prevalence of vitamin D deficiency in children and adolescents aged 6–17 was 53.2% at the cut-off of 20 ng/mL [10]. In the research of the relationship between nutrition and health, young adults are often ignored, including the research on the nutritional status of vitamin D. For example, there are seldom reports focus on vitamin D nutrition status of Chinese college students, and Chinese college freshmen tend to reduce their outdoor sports in the previous year due to the huge pressure of college entrance examination. Considering the non-skeletal health benefits of vitamin D and from the point of view of disease early prevention using nutrition strategy, it is necessary to clarify whether vitamin D deficiency is also a nutritional problem in Chinese college students and its influencing factors.

The main sources of vitamin D in the body are sunlight, diet and vitamin D supplements. Provitamin D (7-dehydrocholesterol, 7-DHC) was converted to previtamin D in the skin by exposure to UVB radiation [11]. Serrano MA reported that around noon in January it took more than two hours of solar exposure to obtain the recommended daily dose of vitamin D, whereas the rest of the year ranged between 7 min on July and 31 min on October in a northern mid-latitude [12]. At moderate to high latitudes, diet became an increasingly important source of vitamin D due to decreased solar intensity and cold temperatures, which discouraged skin exposure [11]. The research of Dimakopoulos I indicated that major food sources of vitamin D were fish (46%), meat (15%) and cereals (12%), however, over 90% of the population in all age groups did not meet the Estimated Average Requirement (EAR) among Greek adults [13]. Some studies also indicated that increased serum vitamin D occurred with physical activity both indoors and outdoors [14]. Most of the studies focused on the separate effects of dietary intake, ultraviolet ray (UV) radiation exposure or physical activity on serum vitamin D levels. But there is not a unique and adequate source of vitamin D for human and vitamin D status is usually the result of a combination of these factors above. Therefore, considering the comprehensive effects of dietary intake, physical activity, and UV protection on vitamin D levels has more practical meaning for an integrated measure.

The purpose of this study was to explore the serum 25(OH)D_3_ status of college freshmen enrolled by Shanghai University of Medicine and Health Science in September 2020, in order to clarify whether vitamin D deficiency is also a health problem for young adults. In addition, the comprehensive effects of diet, physical activity, outdoor time and UV protection on serum 25(OH)D_3_ levels were analyzed, providing theoretical supporting for the comprehensive life-style prevention strategy of vitamin D deficiency.

## Methods

### Participants

The participants of this study were freshmen of ***BLINDED***, started the school in 2020 September. They came from twenty-six different provinces of China. A total of 3573 students recruited into our study. After excluding 113 students who had missing serum 25(OH)D_3_ and 75 students who had incomplete demographic information, A total of 3385 students with completed physical examination and serum 25(OH)D_3_ determination were included in the study. And 1925 students completed the questionnaires on lifestyles. After excluding 257 students who had extreme values for total energy intake (< 500 or > 4500 kcal/d) and 1 case with obvious logic error in data, 1667 subjects were eventually included in our analysis. This study protocol was approved by the Ethics Committee of ***BLINDED*** A written informed consent was provided by all participants.

### Questionnaire survey

An online questionnaire was used to collect the demographic information and the lifestyle information about the diet, physical activity, and UV protection of the participants. The questionnaires were provided in class units and were explained by trained class instructors before the formal investigation. The same IP address can only be answered once. The questionnaire could only be submitted after all the questions were filled out. The first part of the questionnaire was the demographic information, including age, gender, original residence, ethnicity etc. The second part of the questionnaire was a simple food-frequency questionnaire (FFQ) which was designed by Gao J and the reliability and validity were reasonable. A total of 21 food groups that were common consumed in China were included in our FFQ questionnaire. For each food group, the participants were asked how frequently they consumed in the past 12 months, followed by the amount of consumption with five common amounts to choose. The pictures of the standard amount of food were presented when the participants answered questions online. The daily food consumption was calculated by multiplying food frequency by food amount. Before the survey started, 100 participants were recruited to complete our simple FFQ twice with a two-week interval and also finished FFQ146, which included 146 food items produced by Chinese Center for Disease Control and Prevention. The reliability coefficients of food intakes between two simple FFQs ranged from 0.56 to 0.87 (*P* < 0.01). The reliability coefficients of food intakes between the simple FFQ and FFQ146 ranged from 0.31 to 0.72 (*P* < 0.01). The correlation coefficients of food intakes between simple FFQ and 3d 24 h dietary records ranged from 0.37 to 0.65(*P* < 0.01) [15]. The third part of the questionnaire was about the UV protection. The use of UV protection measures including sun hat, sun umbrella, sun-protective clothing and sunscreen was asked. Each question had 5 choices including almost no use, occasional use in summer, frequent use in summer, always use in summer and use in all seasons. For each of the four questions, the choice of almost no use, occasional use in summer, frequent use in summer, always use in summer and use in all seasons was calculated as 1 point, 2 point, 3point, 4 point, and 5 point, respectively. The UV protection index was calculated as the total scores of the four questions. Therefore, the UV protection index ranged from 4 to 20, the higher the score, the better the sun protection. The fourth part of the questionnaire was about the physical activities including the frequency and duration of vigorous physical activities, moderate physical activities and walking every week in the last 1 year. The average duration of physical activities per day was calculated as frequency per week multiplied by duration per time and then divided by 7 days. The average duration of the daytime outdoor activities every day was also asked in this part.

### Anthropometric measurements

Height and weight were obtained by well-trained examiners, with the participants wearing light, thin clothing and no shoes. Body weight and height were measured to the nearest 0.1 kg and 0.1 cm, respectively. Body mass index (BMI) was calculated as weight (kg) divided by the square of the height in meters (m^2^).

### Serum 25(OH)D_3_ determination

Fasting venous blood samples were collected in September and serum 25(OH)D_3_ concentration was measured using a high performance liquid chromatograph (Agilent 1100; Agilent Technologies Inc., Santa Clara, CA, USA) and a mass spectrometer (API4000Q trap; AB SCIEX LLC., Redwood City, CA, USA). The lower limits of 25(OH)D_3_ for detection was 1.6 ng/mL. The test sensitivity was assessed with the inter-batch coefficient of variation (CV) of 5.85% and between batches CV of 6.18%.

There has been a long debate on the cutoff points for vitamin D status. Since the participants are Chinese college students, we use the criteria in Consensus of the Chinese Society of Osteoporosis and Bone Mineral Research: circulating 25(OH)D_3_ < 10 ng/mL was considered severely deficient, 10 ~  < 20 ng/mL deficient, 20 ~  < 30 ng/mL insufficient, and ≥ 30 ng/mL sufficient [16].

### Statistical analyses

SPSS 22.0 was used for statistical analysis. Serum 25(OH)D_3_ concentrations were expressed as P_50_ (P_25_–P_75_). The category variables were expressed as frequency (percentage), Mann–Whitney U test or Kruskal–Wallis H test were used to test differences among groups of serum 25(OH)D_3_ levels. Multiple linear regression analysis was used to screen the dietary factors that influence serum 25(OH)D_3_ levels, adjusted with age, gender and total energy intake. Multiple linear regression analysis was also used to analyze the comprehensive effects of diet, physical activity, and UV protection on serum 25(OH)D_3_ levels, adjusted with age, gender, student’s original residence, taken calcium or vitamin D supplements, latitudes (as continuous variables), longitude (as continuous variables) and BMI categories. Since the distribution of serum 25(OH)D_3_ levels was slightly skewed, the log-transformed serum 25(OH)D_3_ levels was used as the dependent variable in multiple linear regression analysis. A two-sided *P* < 0.05 was considered statistically significant.

## Results

### Basic characteristics of the participants

A total of 1667 participants with completed data were included in this analysis, with 383 males (23.0%) and 1284 females (77.0%). The participants came from twenty-six provinces, autonomous districts or municipalities of China with an average age of 18.6 ± 0.9 years. Levels of serum 25(OH)D_3_ of the participants ranged from 5.2 to 64.8 ng/mL with the mean of 18.1 ± 6.3 ng/mL. There were 67.5% participants had serum 25(OH)D_3_ < 20 ng/mL, 27.8% with the level of 20 ~  < 30 ng/mL and 4.7% with the level of ≥ 30 ng/mL.

Basic characteristics of the participants with serum 25(OH)D_3_ levels were shown in Table 1. The serum 25(OH)D_3_ levels in male was higher than those in female, and participants who came from countryside and town had higher serum 25(OH)D_3_ levels than those who came from city. Participants came from central longitude had relatively higher serum 25(OH)D_3_ levels. And participants who had taken calcium or vitamin D supplements within 3 months had higher serum 25(OH)D3 levels than those without supplements.

**Table 1:** Characteristics of the participants with serum 25(OH)D_3_ levels (n = 1667)

Variables	n %	25(OH)D_3_(ng/mL) ^1^	*P* value ^2^
*Age*			
16–17	31 (1.9)	18.0 (15.7–20.1)	0.32
18–19	1492 (89.5)	17.0 (13.5–21.4)	
20–26	144 (8.6)	17.5 (14.3–22.4)	
*Gender*			
Male	383 (23.0)	20.4 (15.9–25.5)	< 0.001
Female	1284 (77.0)	16.2 (13.0–20.3)	
*Region of original residence*			
City	1072 (64.3)	16.2 (13.1–20.7)	< 0.001
Town	197 (11.8)	18.7 (14.3–22.9)	
Countryside	398 (23.9)	18.4 (14.7–22.7)	
*Ethnicity*			
The Han Ethnic	1591 (95.4)	17.1 (13.5–21.5)	0.70
The Minorities	76 (4.6)	16.7 (13.6–19.9)	
*Taken calcium or vitamin D supplements within 3 months*			
Yes	132 (7.9)	18.8 (15.2–23.3)	0.005
No	1535 (92.1)	17.0 (13.5–21.4)	
*Latitude*			
18° N ~ 24° N	37 (2.2)	19.3 (14.2–26.3)	0.07
25° N ~ 34° N	1437 (86.2)	17.0 (13.4–21.3)	
35° N ~ 44° N	176 (10.6)	18.2 (14.4–21.8)	
45° N ~ 49° N	17 (1.0)	17.6 (13.5–24.2)	
*Longitude*			
76° E ~ 94° E	36 (2.2)	15.6 (12.9–18.7)	< 0.001
95° E ~ 114° E	241 (14.5)	19.0 (15.2–23.1)	
115° E ~ 131° E	1390 (83.4)	16.8 (13.3–21.2)	
*BMI categories*^*3*^			
< 18.5	390 (23.4)	16.3 (13.1–20.5)	0.001
18.5 ~ < 24.0	906 (54.3)	17.1 (13.6–21.2)	
24.0 ~ < 28.0	225 (13.5)	18.1 (14.1–23.1)	
≥ 28.0	86 (5.2)	18.0 (14.5–22.3)	

^1^ Serum 25(OH)D_3_ levels were expressed as P_50_ (P_25_–P_75_)

^2^ *P* value for the Mann–Whitney U test or Kruskal–Wallis H test

^3^ 60 data missing in variable BMI

### Serum 25(OH)D_3_ levels of the participants with different food intake amount

The participants were divided into two groups according to the common daily intake amount of each food group, high intake group and low intake group. The serum 25(OH)D_3_ levels of the participants with different food intake amount were shown in Table 2. Participants in high intake groups of refined rice, whole grains, milk and yogurt, eggs, river fish, shrimp and crab had higher serum 25(OH)D_3_ levels than those in low intake groups. However, participants in high intake group of candy had lower serum 25(OH)D_3_ levels than those in low intake group. And participants who had taken calcium or vitamin D supplements within 3 months had higher serum 25(OH)D_3_ levels than those without supplements.

**Table2:** Serum 25(OH)D_3_ levels of the participants with different food intake amount (n = 1,667)

Food items	N(%)	25(OH)D_3_(ng/mL)^1^	*P* value^2^
*Refined rice, steamed*			
< 200 g/d	568 (34.1)	16.3 (13.0–20.8)	0.006
≥ 200 g/d	1099 (65.9)	17.3 (13.8–21.7)	
*Refined rice, porridge*			
< 50 g/d	1010 (60.6)	17.0 (13.5–21.4)	0.24
≥ 50 g/d	657 (39.4)	17.3 (13.6–21.5)	
*Refined wheat products*			
< 50 g/d	707 (42.4)	17.1 (13.4–21.3)	0.58
≥ 50 g/d	960 (57.6)	17.0 (13.6–21.6)	
*Desserts*			
< 25 g/d	926 (55.5)	17.4 (13.6–22.0)	0.19
≥ 25 g/d	741 (44.5)	16.7 (13.5–21.1)	
*Whole grains*			
< 25 g/d	1072 (64.3)	17.0 (13.5–21.1)	0.048
≥ 25 g/d	595 (35.7)	17.4 (13.6–22.3)	
*Tubers*			
< 25 g/d	942 (56.5)	17.0 (13.6–21.2)	0.62
≥ 25 g/d	725 (43.5)	17.3 (13.5–21.7)	
*Milk and yogurt*			
< 200 mL/d	800 (48.0)	16.5 (13.0–20.9)	0.001
≥ 200 mL/d	867 (52.0)	17.4 (13.8–22.0)	
*Eggs*			
< 50 g/d	1080 (64.8)	16.7 (13.3–21.2)	0.01
≥ 50 g/d	587 (35.2)	17.6 (14.0–22.0)	
*Livestock meat*			
< 100 g/d	820 (49.2)	16.9 (13.5–21.4)	0.29
≥ 100 g/d	847 (50.8)	17.1 (13.6–21.5)	
*Poultry meat*			
< 50 g/d	823 (49.4)	17.0 (13.6–21.2)	0.62
≥ 50 g/d	844 (50.6)	17.1 (13.5–21.7)	
*River fish, shrimp and crab*			
< 25 g/d	1151 (69.0)	16.8 (13.4–21.2)	0.03
≥ 25 g/d	516 (31.0)	17.5 (13.9–22.2)	
*Sea fish, shrimp and crab*			
< 25 g/d	1347 (80.8)	17.0 (13.5–21.2)	0.34
≥ 25 g/d	320 (19.2)	17.1 (13.9–22.0)	
*Soybeans and Soy products*			
< 25 g/d	877 (52.6)	16.8 (13.2–21.1)	0.06
≥ 25 g/d	790 (47.4)	17.3 (13.8–21.7)	
*Nuts and seeds*			
< 10 g/d	1004 (60.2)	17.1 (13.6–21.1)	0.60
≥ 10 g/d	663 (39.8)	16.9 (13.4–22.0)	
*Dark vegetables*			
< 100 g/d	881 (52.8)	16.8 (13.6–21.1)	0.08
≥ 100 g/d	786 (47.2)	17.5 (13.5–22.0)	
*Light color vegetables*			
< 50 g/d	710 (42.6)	16.8 (13.5–21.1)	0.17
≥ 50 g/d	957 (57.4)	17.3 (13.6–21.8)	
*Mushrooms*			
< 25 g/d	1053 (63.2)	17.0 (13.6–21.3)	0.69
≥ 25 g/d	614 (36.8)	17.2 (13.5–21.7)	
*Fruits*			
< 100 g/d	832 (49.9)	17.0 (13.4–21.2)	0.18
≥ 100 g/d	835 (50.1)	17.1 (13.6–21.6)	
*Puffed foods*			
< 25 g/d	1269 (76.1)	17.3 (13.7–21.5)	0.08
≥ 25 g/d	398 (23.9)	16.5 (13.0–21.2)	
*Candy*			
< 10 g/d	926 (55.5)	17.5 (13.7–22.0)	0.007
≥ 10 g/d	741 (44.5)	16.4 (13.2–21.0)	
*Sugary drinks*			
< 100 mL/d	910 (54.6)	17.4 (13.7–21.4)	0.12
≥ 100 mL/d	757 (45.4)	16.5 (13.3–21.6)	
*Took calcium or vitamin D supplements within 3 months*			
Yes	132 (7.9)	18.8 (15.2–23.3)	0.005
No	1535 (92.1)	17.0 (13.5–21.4)	

^1^ Serum 25(OH)D_3_ levels were expressed as P_50_ (P_25_–P_75_)

^2^ *P* value for the Mann–Whitney U test

### Association of dietary factors with serum 25(OH)D_3_ levels

Multivariate linear regression analysis indicated that milk and yogurt intake, calcium or vitamin D supplementation were significantly associated with higher serum 25(OH)D_3_ levels and candy intake was reversely associated with serum 25(OH)D_3_ levels, adjusted for age, gender and total energy intake, as shown in Table 3.

**Table3:** Multivariate linear regression analysis on the association of dietary factors with serum 25(OH)D_3_ levels (n = 1,667)

Food items	*β* value	Standard *β* value	*P* value
*Refined rice, steamed*			
< 200 g /d	Ref	Ref	Ref
≥ 200 g /d	0.03	0.04	0.09
*Refined rice, porridge*			
< 50 g /d	Ref	Ref	Ref
≥ 50 g /d	0.05	0.02	0.34
*Refined wheat products*			
< 50 g /d	Ref	Ref	Ref
≥ 50 g /d	− 0.01	− 0.02	0.47
*Desserts*			
< 25 g /d	Ref	Ref	Ref
≥ 25 g /d	− 0.007	− 0.01	0.72
*Whole grains*			
< 25 g /d	Ref	Ref	Ref
≥ 25 g /d	0.02	0.03	0.35
*Tubers*			
< 25 g /d	Ref	Ref	Ref
≥ 25 g /d	− 0.005	− 0.007	0.77
*Milk and yogurt*			
< 200 mL /d	Ref	Ref	Ref
≥ 200 mL /d	0.05	0.08	0.002
*Eggs*			
< 50 g /d	Ref	Ref	Ref
≥ 50 g /d	0.01	0.02	0.45
*Livestock meat*			
< 100 g /d	Ref	Ref	Ref
≥ 100 g /d	− 0.001	− 0.002	0.94
*Poultry meat*			
< 50 g /d	Ref	Ref	Ref
≥ 50 g /d	− 0.01	− 0.02	0.46
*River fish, shrimp and crab*			
< 25 g /d	Ref	Ref	Ref
≥ 25 g /d	0.03	0.04	0.14
*Sea fish, shrimp and crab*			
< 25 g /d	Ref	Ref	Ref
≥ 25 g /d	− 0.02	− 0.03	0.37
*Soybeans and Soy products*			
< 25 g /d	Ref	Ref	Ref
≥ 25 g /d	0.01	0.02	0.50
*Nuts and seeds*			
< 10 g /d	Ref	Ref	Ref
≥ 10 g /d	− 0.01	− 0.02	0.55
*Dark vegetables*			
< 100 g /d	Ref	Ref	Ref
≥ 100 g /d	0.001	0.002	0.95
*Light color vegetables*			
< 50 g /d	Ref	Ref	Ref
≥ 50 g /d	− 0.003	− 0.005	0.86
*Mushrooms*			
< 25 g /d	Ref	Ref	Ref
≥ 25 g /d	− 0.02	− 0.03	0.25
*Fruits*			
< 100 g /d	Ref	Ref	Ref
≥ 100 g /d	0.03	0.05	0.07
*Puffed foods*			
< 25 g /d	Ref	Ref	Ref
≥ 25 g /d	0.003	0.004	0.87
*Candy*			
< 10 g /d	Ref	Ref	Ref
≥ 10 g /d	− 0.04	− 0.05	0.048
*Sugary drinks*			
< 100 mL /d	Ref	Ref	Ref
≥ 100 mL /d	− 0.03	− 0.04	0.16
*Taken calcium or vitamin D supplements within 3 months*			
No	Ref	Ref	Ref
Yes	0.07	0.05	0.03

Multivariate linear regression analysis model was used, adjusted for age, gender and total energy intake

### Serum 25(OH)D_3_ levels in different UV protection categories

**Fig. 1:**
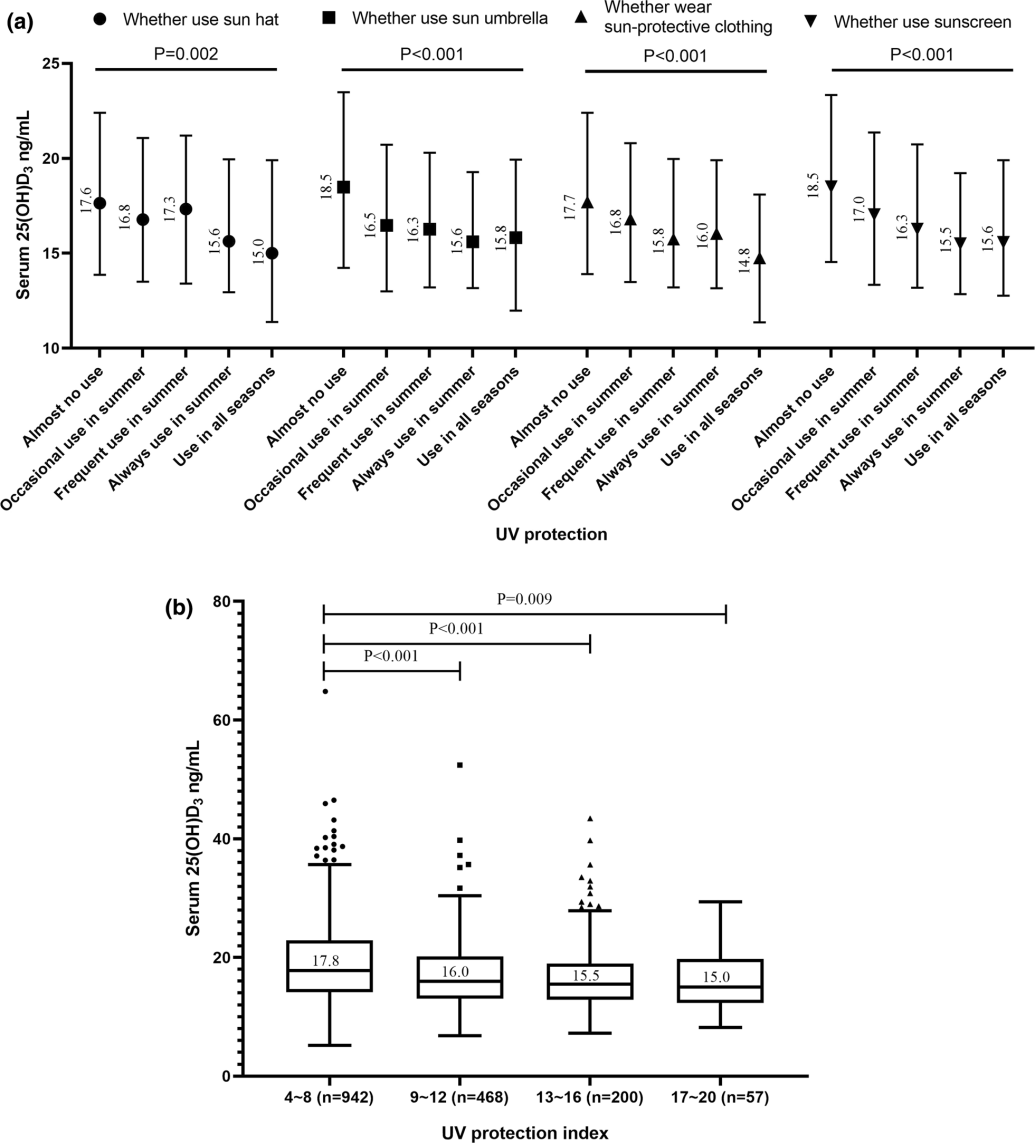
**A** Serum 25(OH)D_3_ levels in different UV protection categories (n = 1667). **B** Serum 25(OH)D_3_ levels in different UV protection index categories (n = 1667)

Among the participants, 79.8% used at least one kind of the UV protection measure. Serum 25(OH)D_3_ levels in different UV protection categories were shown in Fig. 1A. Participants with higher UV protection frequency tended to have lower serum 25(OH)D_3_ levels levels, no matter which type of UV protection measure was used (*P* < 0.05). And serum 25(OH)D_3_ levels decreased with the increase of the UV protection index as shown in Fig. 1B.

### Serum 25(OH)D_3_ levels in different physical activity

Serum 25(OH)D_3_ levels in physical activity were shown in Table 4. Participants who had outdoor activity time ≥ 1 h/day had higher serum 25(OH)D_3_ levels than others.

**Table 4:** Serum 25(OH)D_3_ levels in different physical activity categories (n = 1667)

Variables	N (%)	25(OH)D_3_(ng/mL)^1^	*P* value^2^
*Total hours of vigorous physical activity per week*			
< 1 h/day	648(38.9)	17.0 (13.3–20.8)	< 0.001
1 ~ < 2 h/day	543(32.6)	16.4 (13.2–21.3)	
≥ 2 h/day	476(28.6)	17.8 (14.4–22.8)	
*Total hours of moderate physical activity per week*			
< 1 h/day	994(59.6)	17.0 (13.5–21.5)	0.44
1 ~ < 2 h/day	406(24.4)	17.2 (13.7–21.6)	
≥ 2 h/day	267(16.0)	17.0 (13.2–21.4)	
*Average walking time per day*			
< 0.5 h/day	588(35.3)	17.7 (13.9–22.4)	0.19
0.5 ~ < 1 h/day	622(37.3)	16.8 (13.5–20.8)	
≥ 1 h/day	457(27.4)	15.8 (13.2–20.0)	
*Average time of outdoor activity per day*			
< 0.5 h/day	517(31.0)	16.4 (13.1–20.7)	< 0.001
0.5 ~ < 1 h/day	527(31.6)	16.2 (12.9–20.8)	
≥ 1 h/day	623(37.4)	18.0 (14.6–22.6)	

^1^Serum 25(OH)D_3_ levels were expressed as P_50_ (P_25_–P_75_)

^2^*P* value for the Mann–Whitney U test or Kruskal–Wallis H test

### Association of diet, physical activity and UV protection with serum 25(OH)D_3_ levels

Multiple regression analysis showed that higher intake of milk and yogurt, taken calcium or vitamin D supplementation, and longer time of outdoor activity were significantly associated with higher serum 25(OH)D_3_ levels, while higher candy intake, higher UV protection index were significantly associated with lower serum 25(OH)D_3_ levels, after adjusted for age, gender, students’ original residence, latitudes, longitude and BMI (*P* < 0.05) as shown in Table 5. There were no interactions among diet factors, outdoor activity time and UV protection index (data not shown).

**Table 5:** Association of diet, physical activity and UV protection on serum 25(OH)D_3_ levels (n = 1667)

Variables	*β* value	Standard *β* value	*P* value
*Milk and yogurt intake*			
< 200 mL/d	Ref	Ref	Ref
≥ 200 mL/d	0.05	0.08	0.001
*Candy intake*			
< 10 g/d	Ref	Ref	Ref
≥ 10 g/d	− 0.04	− 0.06	0.01
*Taken calcium or vitamin D supplements within 3 months*			
No	Ref	Ref	Ref
Yes	0.07	0.05	0.02
*Total hours of vigorous physical activity per week*			
< 1 h/day	Ref	Ref	Ref
1 ~ < 2 h/day	− 0.009	− 0.01	0.63
≥ 2 h/day	0.01	0.02	0.60
Average time of outdoor activity per day			
< 0.5 h/day	Ref	Ref	Ref
0.5 ~ < 1 h/day	0.01	0.02	0.50
≥ 1 h/day	0.07	0.10	< 0.001
UV protection index	− 0.006	− 0.06	0.02

Multivariate linear regression analysis model was used, adjusted for age, gender, students’ original residence, latitudes, longitude and BMI categories

## Discussion

In this study, the vitamin D status and its possible influencing factors including diet, physical activity, and UV protection were explored in 1667 college freshmen who came from 26 different provinces of China for the first time. The mean serum 25(OH)D_3_ levels were 18.1 ng/mL and the prevalence of deficiency and insufficiency was 67.5% and 27.8% respectively even at the end of summer. Among the modifiable factors that commonly influence serum 25(OH)D_3_ status, higher intake of milk and yogurt, taken calcium or vitamin D supplementation, and longer time of outdoor activity were significantly associated with higher serum 25(OH)D_3_ levels, while higher candy intake, higher UV protection index were significantly associated with lower serum 25(OH)D_3_ levels, which collectively affect serum 25(OH)D_3_ levels.

Studies have shown that vitamin D deficiency is still widespread around the world [7, 8]. Most of the research focus on children, pregnant women and old people but it is relatively limited in young adults [9, 17–19]. Nimri LF reported that vitamin D deficiency reached 47.9%, and the mean 25(OH)D_3_ levels were 21.7 ng/mL among US female college students [20]. The mean serum 25(OH)D_3_ levels was 18.7 ng/mL in our study, and the mean serum 25(OH)D_3_ levels were 17.2 ng/mL in female which was much lower than the US college students, and also lower than that in the elderly Chinese population (24.4 ng/mL) [21]. Our results indicated that vitamin D deficiency and insufficiency were serious in Chinese college students. Since serum 25(OH)D_3_ status was influenced by ethnicity, geographical location, season, outdoor activity, diet and UV protection, our study focused on modifiable factors such as outdoor activity, diet and UV protection to explore the feasibility of preventing vitamin D deficiency from a lifestyle perspective.

Vitamin D is synthesized in the skin but can also be obtained from foods and supplements. However, there are very few natural food sources rich in vitamin D [22]. In our study, only milk and yogurt had positive association with serum 25(OH)D_3_ in the natural food, whereas the clinical effect was very limited with the difference only 0.9 ng/mL between group < 200 mL/d and group ≥ 200 mL/d. Some studies had shown that milk consumers consistently had higher serum 25(OH)D_3_ levels [23, 24]. The results of the US National Health and Nutrition Examination Survey (NHANES 2001–2010) demonstrated a significant association between milk consumption and serum vitamin D status in US population, and the probability of meeting vitamin D recommendations was greater in milk consumers vs. non-consumers [23]. The reason may be that almost all milk in the US was fortified with 100 IU/cup vitamin D irrespective of the type of milk. In China, not all milk was fortified with vitamin D. Therefore, in our study, although milk and yogurt had an independent influence on serum 25(OH)D_3_ levels, the clinical effect was very limited. Systematic vitamin D food fortification is, however, an effective approach to improve vitamin D status in the general population [25]. WHO-FAO suggest that food fortification tends to have a less immediate but nevertheless a much wider and more sustained impact. Furthermore, as the benefits are potentially large, food fortification can be a very cost-effective public health intervention [26]. Therefore, it is necessary for our country to make legislation or food standards that provide guidance or regulations for fortification of food with vitamin D on a voluntary or mandatory basis. The Dietary Guidelines for Chinese Residents [27] recommended a variety of dairy products, equivalent to 300 g of liquid milk per day. However, in our study, only 25% of the participants met the recommendation for milk. Therefore, vitamin D food fortification and encouraging milk intake may be an effective strategy to improve the vitamin D status for Chinese college students.

Another dietary factor which had independent influence on serum 25(OH)D_3_ levels was candy intake in our study. The candy intake ≥ 10 g/d group was significantly associated with lower serum 25(OH)D_3_ levels in Chinese college students. A monitored data from Korea National Health and Nutrition Examination Survey (KNHANES) had observed an increasing trend of consume sugary drinks and have vitamin D deficiencies in children and teenagers [28]. So far the study about the relationship between candy or sugar intake and serum vitamin D status is limited. But the current evidence suggested a reverse association of candy or sugar intake with serum 25(OH)D_3_ status, which needs to be further clarified in detail.

Since very few foods naturally contain vitamin D, the intake of vitamin D from foods is very low worldwide and well below the EAR [13, 29]. Therefore, vitamin D supplementation sometimes is considered a strategy to improve vitamin D deficiency. Numerous agencies and scientific organizations reached a consensus that serum 25(OH)D_3_ concentrations of 30–50 ng/mL are beneficial for overall health and in the absence of regular sun exposure, using appropriate doses of vitamin D supplements is the most efficient way to improve serum vitamin D status [30]. However, the current vitamin D supplement guidelines in China only apply to infants, women in late pregnancy or the prevention of osteoporosis [31, 32], and there is no guideline on vitamin D supplement for “healthy” young people in China. American association of clinical endocrinologist medical guidelines suggested that low bone mass and skeletal fragility in adults may be the result of low peak bone mass in early adulthood, excessive bone loss in later life, or both [33]. Whereas peak adult bone mass is usually attained in the late teens or early 20 s [34]. Therefore, the concern of vitamin D nutrition in young adults is very important for skeletal health, which is usually neglected currently. Vitamin D is also associated with non-skeletal diseases, including cardiovascular disease, metabolic syndrome (MetS) and cancer [1–3]. And health conditions in early life including childhood and youth, would influence risks of disease in middle and old age [35]. These collectively suggest that maintaining adequate vitamin D levels in early adulthood can help prevent these skeletal and non-skeletal diseases later in life, and it is important for us to clarify the nutritional status of vitamin D in college students. In our study, only 4.7% participants met the recommendation of serum 25(OH)D_3_ concentrations 30–50 ng/mL, and only 7.9% participants took calcium or vitamin D supplements, and participants who took calcium or vitamin D supplements had higher serum 25(OH)D_3_ levels in our study. The WHO-FAO suggest that supplementation is often the fastest way to control deficiency in population groups because of the advantage of being capable of supplying an optimal amount of a specific nutrient and in a highly absorbable form [26]. Therefore, we think it is necessary to consider formulating corresponding vitamin D supplementation programs for young adults, in condition of the sun exposure and dietary intake is not enough.

On the global level, the main source of vitamin D is the sun[11]. However, UV radiation is a double-edged sword, excessive UV exposure leads to skin cancer while lack of UV exposure leads to vitamin D deficiency [36]. More and more people take measures against UV exposure nowadays [37]. In this study, 79.8% of the participants used at least one kind of the UV protection measure and we used the UV protection index to assess the comprehensive degree of the participants’ UV protection. Participants with higher UV protection index tended to have lower serum 25(OH)D_3_ levels and serum 25(OH)D_3_ levels decreased with the increase of the UV protection index. Since the college students especially female at this age in China usually pay more attention to UV protection, it is necessary to develop a well-balanced guideline on UV protection that ensure an adequate vitamin D status and also skin protection.

The relationship between physical activity and serum vitamin D has also been concerned by some researchers. The findings of the analysis based on data from the US NHANES indicated that physical activity was associated with high levels of serum 25(OH)D_3_ regardless of indoor or outdoor practice, but the association was stronger in outdoor compared with indoor environments [14, 38]. In this study, total hours of physical activity per week was not an independent factor for serum 25(OH)D_3_ levels, whereas longer time of outdoor activity was significantly associated with higher serum 25(OH)D_3_ levels. Therefore, the relationship between outdoor activity and serum 25(OH)D_3_ levels is still attributed to the UV exposure of Chinese college students. Spending more than one hour a day for outdoor activity should be encouraged for the aim of increasing serum 25(OH)D_3_ levels in Chinese college students according to our study.

The strength of our study was that the participants of our study came from 26 different provinces which covered a wide geographical range of China, therefore, the sample was well representative. Taking college students as research subjects, we comprehensively analyze the association of diet, physical activity, and UV protection with Vitamin D status, thus filling the research blank of vitamin D deficiency in related fields in China. There were still some limitations in our study. First, the information of diet, physical activity, and UV protection were got from the online questionnaire filled out by students themselves which may lead to selection bias, although we had provided filling explanation and training before investigation. Second, the result came from the cross-section study, although we adjusted the common confounding factors in the multivariate linear regression, unknown confounding factors are still hard to avoid. The causal association of diet, physical activity and UV protection with serum 25(OH)D_3_ status needs to be clarified by further prospective studies.

## Conclusions

The serum 25(OH)D_3_ status of the college students in China was not optimistic even after the summer vacation. This may be attributed to the heavy academic stress and the lack of outdoor sports among Chinese students. Therefore, it is necessary to develop methods and strategies to improve the serum 25(OH)D_3_ status of Chinese college students. Milk and yogurt intake and outdoor activity should be encouraged, while candy intake should be limited in Chinese college students for the aim of preventing vitamin D deficiency. Public health policies should consider a well-balanced guideline on UV protection. At the same time, whether vitamin D food fortification or vitamin D supplements could be considered to ensure an adequate vitamin D status in young adults should be further discussed.

## Data Availability

Data generated or analyzed during this study are included in this article and are available from the corresponding author on reasonable request.
